# Programmable DNA-Based Dimerization Networks for Dynamic and Competitive Control of Transcription

**DOI:** 10.1021/jacs.6c02173

**Published:** 2026-07-20

**Authors:** Miriam Quattrociocchi, Simone Brannetti, Erica Del Grosso, Francesco Ricci

**Affiliations:** Department of Chemical Sciences and Technologies, University of Rome, Tor Vergata, Via della Ricerca Scientifica, Rome 00133, Italy

## Abstract

Here, we introduce a minimal, experimentally validated DNA-encoded analogue of many-to-many protein dimerization networks to achieve programmable control of cell-free transcription. To do so, we rationally designed DNA monomers bearing orthogonal azide or DBCO handles that assemble via SPAAC chemistry into a combinatorial library of covalent dimers where only a single dimer completes an otherwise inactive promoter and activates transcription of a light-up RNA aptamer. By expanding the network from 2 to 22 monomers, we show that the RNA yield can be finely tuned over more than an order of magnitude, in quantitative agreement with a simple combinatorial model in which the activator dimer fraction decreases as a function of network size. We also designed sequence-defined inputs that selectively sequester monomers to reduce network size and upregulate chosen dimer activators, enabling multiplexed, orthogonal activation of different templates in the same solution from a shared monomer pool. Coupling the dimerization layer to a CRISPR–Cas12a collateral cleavage module, we further show that competitive DNA dimerization transcription logic can be interfaced with downstream enzymatic reactions to convert input sets into amplified cleavage activity. Together, these results establish competitive covalent DNA dimerization networks as a modular and quantitatively predictable platform for implementing competitive many-to-many dimerization logic for controlling cell-free transcription outputs.

## Graphical abstract

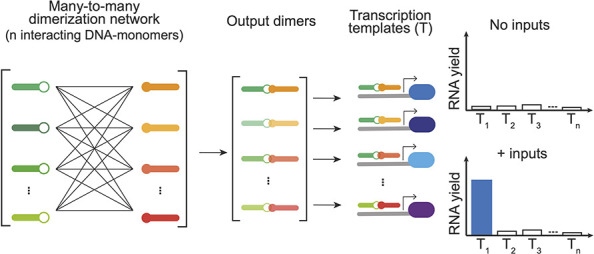

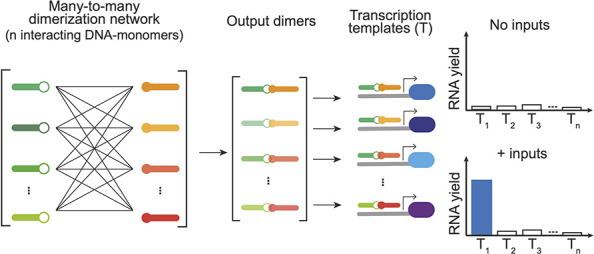

## Introduction

Natural biological systems frequently rely on dimerization-based regulatory networks to achieve sophisticated control over gene expression and cellular decision-making.^1–5^ Transcription factors, for instance, often function as homo- or heterodimers whose formation, dissociation, and competitive partner choice finely tune promoter occupancy and transcriptional output.^6–8^ Classic examples include basic leucine zipper,^1–9^ helix–loop–helix,^10,11^ and nuclear receptor families,^12,13^ where dimerization controls processes such as developmental patterning, hormone sensing, and stress responses.^14^ In these systems, competitive binding between alternative dimer pairs, cooperative assembly on DNA, and sequestration by inactive partners collectively expand the dynamic range and logic of regulatory responses converting relatively simple protein–protein and protein–DNA interactions into rich input–output behaviors.^4,5^

Inspired by these natural dimerization-based circuits, synthetic biology has sought to recreate and extend similar network architectures in the lab to understand and use similar strategies for cellular control.^15–17^ A minimal circuit design, termed MultiFate, for example, has been developed using a set of engineered zinc-finger transcription factors that can promiscuously homo- and heterodimerize creating stable, controllable cell fates that can switch with external signals, forming a basis for complex synthetic biology.^18^ Protein dimerization modules have been also used to create programmable, multi-input synthetic gene circuits in mammalian cells that act as AND/OR logic gates, allowing precise control over gene expression based on multiple small-molecule inputs and achieving high signal-to-noise ratios for complex synthetic computations.^19,20^ While these protein-based systems showcase the power of dimerization networks for implementing rich regulatory behaviors, they often require elaborate protein engineering and cell-based optimization, which can limit their modularity, scalability, and adaptability in different contexts.

In parallel, nucleic acid–based systems—including DNA strand-displacement reactions and DNA networks—have been used to build synthetic genetic circuits and regulatory modules to program and control gene transcription and translation in cell-free systems.^21–24^ Thanks to the high versatility and predictability of DNA–DNA interactions, DNA nanotechnology in fact appears particularly well suited to emulate biochemical networks because it permits precise control over valency, geometry, and interaction strengths and could be successfully applied to construct synthetic genetic circuits and regulatory modules.^25–31^ Despite this, however, the computational abilities of nucleic acid based networks are still far from those of their protein-based counterparts. Motivated by these considerations, we focus here on establishing a minimal and quantitatively predictable physical implementation of competitive many-to-many dimerization using nucleic acids. To do so, we designed DNA-based dimerization networks employing covalent strain-promoted azide–alkyne cycloaddition (SPAAC) chemistry^32^ to enable precise, tunable control over cell-free transcription reactions through input-dependent modulation of DNA dimer formation. In our system, the competition is encoded at the level of the monomer network, so that adding or removing monomer species controls many potential dimers without redesigning individual strand-displacement reactions. In this work, we implement and quantitatively characterize minimal subnetworks of a competitive many-to-many dimerization architecture, rather than an exhaustive enumeration of all possible dimers. The experimentally accessible subnetworks are chosen to be representative of the broader design space and are interpreted through a simple combinatorial model that captures how network size and composition control transcriptional outputs. By integrating competitive covalent DNA dimer networks with transcriptional control, our work introduces a versatile platform that expands the molecular toolkit for synthetic biology, facilitating dynamic regulation, signal integration, and multiplexed control in cell-free systems with simplicity and programmability not previously achieved.

## Results and Discussion

Our synthetic, input-responsive DNA-based dimerization network consists of a set of single-stranded DNA oligonucleotides monomers (M_i_) each modified at one end with a reactive moiety (either at the 5′-end or at the 3′-end) (Figure 1a). In our system, two distinct reactive groups are used, and each DNA monomer carries only one of these two groups. Consequently, every monomer bearing reactive group #1 can, in principle, react with any monomer bearing reactive group #2, giving access to a many-to-many network configuration and a combinatorial library of covalent DNA dimers. In this library, the network size (*N*) defines the total number of distinct monomer species present in the dimerization reaction. Each possible dimer output could be wired to a distinct incomplete transcription template, thereby activating that template and triggering transcription of a specific RNA strand encoded downstream (Figure 1a). We note, however, that in this work, we experimentally addressed only a small subset of dimers, each connected to one or two templates as described below, thus realizing well-defined subnetworks within the broader combinatorial space of possible dimers. The yield of each of the possible DNA dimers (and thus of the corresponding transcribed RNA) produced by this network will depend on its overall size and will decrease as the network size increases, since all the monomers modified with a certain reactive group are able to indistinctly react with all the monomers modified with the other reactive group (Figure 1b). The addition of specific DNA input strands that are able to sequester defined monomers excluding them from the reaction mixture leads to a reduced network size and to a selective upregulation of a specific dimer output, thus inducing the increase in the yield of a selected RNA strand (Figure 1c).

**Figure 1:**
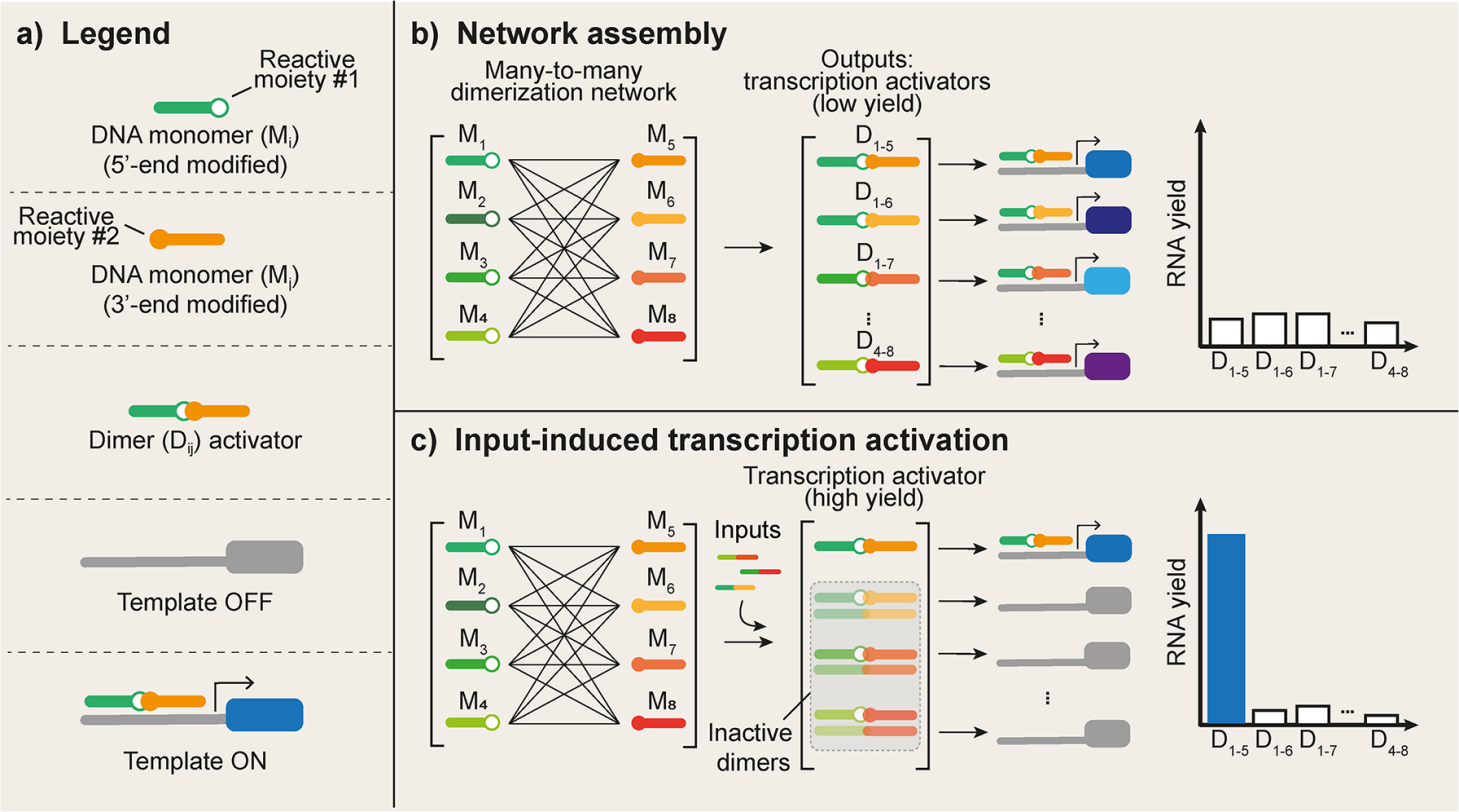
Programmable DNA dimerization network for input-induced transcription activation. (a) Network components. (b) Network assembly. A set of DNA monomers (M) bearing orthogonal reactive moieties at their 5′ and/or 3′-ends is combined to generate a many-to-many dimerization network that yields a low basal population of DNA dimers (D). Each dimer presents a specific sequence that can switch an OFF transcription template to the ON state, producing a specific RNA output. (c) Input-induced transcription activation. Addition of defined DNA inputs selectively sequester a subset of monomers, redistributing the network toward a specific transcription-competent dimer. This results in enhanced RNA production from a selected template, while templates that are not addressed remain inactive and produce only background RNA levels.

To demonstrate transcriptional control through a competitive dimerization network, we have employed in this work azide-modified and DBCO-modified DNA monomers that, through the SPAAC reaction, can form dimer outputs (Figure 2a). The DNA monomers have lengths between 9 and 17 nucleotides (nt) (see the Supporting Information) and each of them has a specific and orthogonal sequence. First, we confirmed the successful covalent assembly of a single dimer (D_1–2_) through a SPAAC reaction carried out under equimolar concentrations (i.e., 1.0 μM) of two monomers (M_1_ + M_2_). Ion-exchange chromatography and gel electrophoresis reveal distinct chromatographic peaks and bands, respectively, for monomers and dimers (Figure 2b, Figure S1). We then demonstrated the transcription activation of an incomplete template (at a concentration of 100 nM) designed to be specifically activated by such a dimer and encoding for the 46-nt light-up mango aptamer (Figure 2c).^33^ Quantitative fluorescence data demonstrate robust activation, with substantially increased signal output of the mango RNA aptamer in the presence of the activator dimer versus monomer controls (Figure 2d,e). Dimer-induced template activation is concentration-dependent with the plateau reached at ca. 10^–8^ M concentrations of both monomers and with an observable *K*_1/2_ (here defined as the monomers’ concentration giving half of the maximum RNA yield) of 3.5 ± 0.4 × 10^–8^ M (Figure 2f).

**Figure 2:**
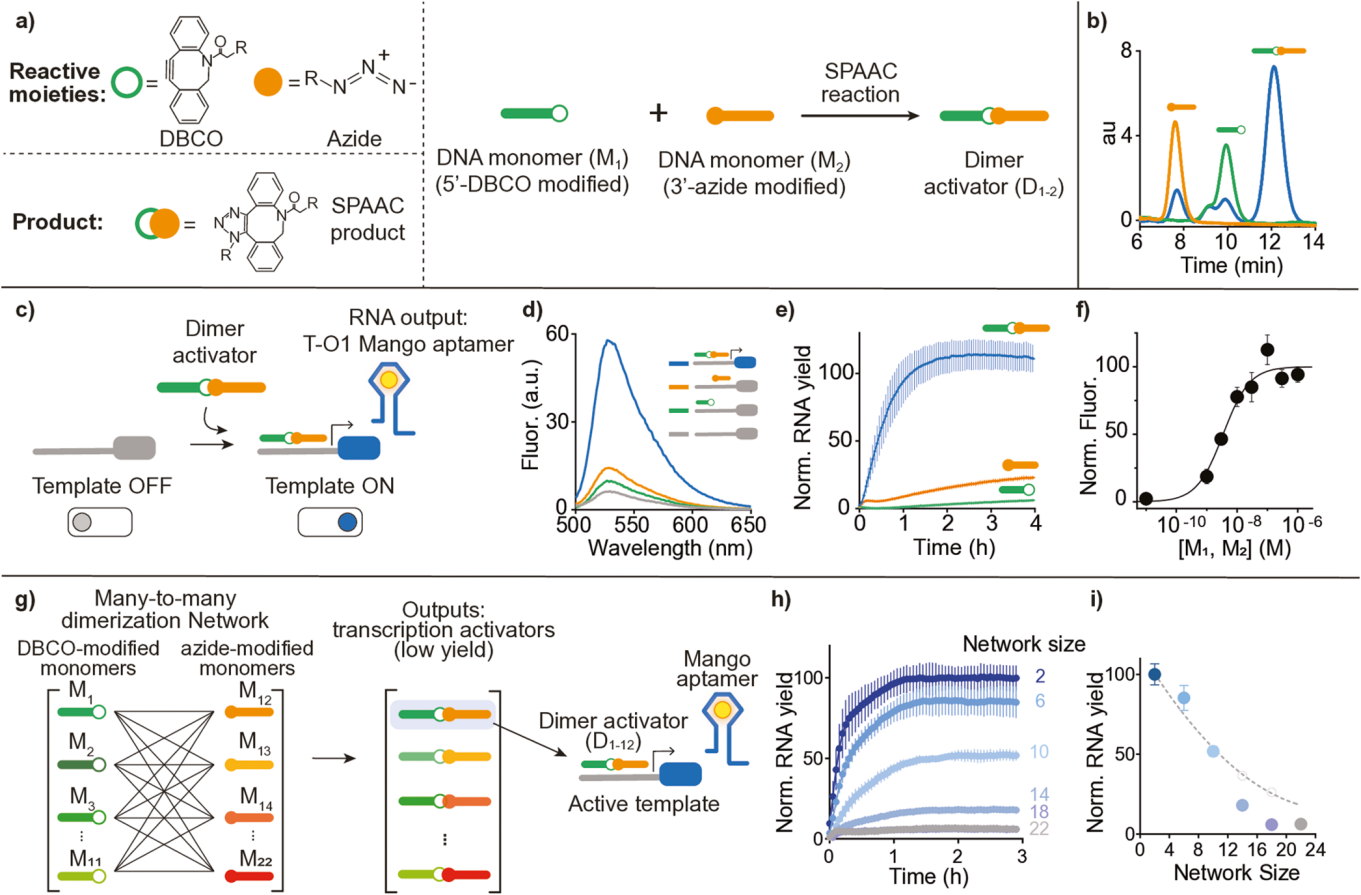
Assembly and characterization of a many-to-many dimerization network for transcription control. (a) DBCO–azide SPAAC reaction used to assemble DNA dimers (D_1–2_) from 5′-DBCO-modified DNA monomers (M_1_) and 3′-azide-modified DNA monomers (M_2_). (b) Ion-exchange chromatography shows the formation of the covalent DNA dimer product (blue trace) from the two DNA monomer precursors (orange and green traces). (c) Schematic of a dimer activator that switches a transcription template from OFF to ON, enabling T7-driven transcription of a mango aptamer RNA output. (d) End point fluorescence emission spectra of the mango aptamer reporter in the presence of the DNA dimer activator, and the two control monomers confirming transcription activation only with the dimer. (e) Time course of normalized RNA yield for the dimer activator and single monomers. (f) Normalized RNA mango fluorescence signal as a function of the monomers’ concentration (always assuming an equimolar concentration between M_1_ and M_2_), obtained from plateau values of the kinetic traces. (g) Scheme of the many-to-many DNA dimerization network in which multiple DNA monomers combinatorially assemble into a pool of DNA dimer transcription activators with distinct sequence compositions. (h) Time courses of normalized RNA yield for networks of increasing size. (i) Normalized RNA yield (at end point) as a function of network size. In the same plot is also shown the theoretical curve obtained from the simple combinatorial model (eqs 1 and 2) using the experimentally determined *K*_1/2_ from panel (f) (dotted gray line). The experiments shown in this figure were performed in 40 mM Tris-HCl, 30 mM MgCl_2_, 10 mM DTT, and 6 mM spermidine in DEPC water, pH 7. Each azide- and DBCO–DNA monomer was mixed at a concentration of 0.5 μM. At the end of each reaction, the network solution was diluted 1:10 in a transcription buffer solution (40 mM Tris-HCl, 30 mM MgCl_2_, 10 mM DTT, 6 mM spermidine, 3 mM nucleoside triphosphate (NTPs), 3.0 U/μL T7 RNA polymerase, 1 U/μL RiboLock, pH 7.0) containing the incomplete template (100 nM) and the mango aptamer dye (TO-1, 200 nM). The transcription reaction was allowed to proceed at 37 °C and fluorescence measured continuously. All reported RNA yields in this and in the following figures correspond to normalized fluorescence units and do not represent absolute RNA concentrations. Error bars represent the standard deviation based on triplicate measurements.

We then proceeded to assemble many-to-many dimerization competitive networks of increasing size by designing different orthogonal azide- and DBCO-modified monomers forming a library of activator dimers. Network size was varied from 2 (1 azide- and 1 DBCO-modified monomer) to 22 (11 azide- and 11 DBCO-modified monomers) by adding equimolar concentrations (1.0 μM) of each monomer and monitoring the transcription yield of the mango aptamer producing template (Figure 2g). Of note, the network was kept “symmetric” by using equal numbers of azide- and DBCO-modified monomers. As expected, as the network size increases, the template activation efficiency and thus the resultant normalized transcriptional signal output decreases from 100 ± 6% (network size = 2) to 6.3 ± 0.4 (network size = 22), confirming that the system enables predictable modulation of cell-free transcription through DNA network assembly (Figure 2h,i).

In developing our combinatorial model, we hypothesize that SPAAC dimerization yields are approximately sequence-independent for the 9–17 nt oligonucleotides and compositions used here. This assumption is supported by ion-exchange chromatography of different distinct azide/DBCO monomer pairs with different lengths and sequences, which all reach similar dimer/monomer ratios under our standard conditions (Figure S2).

To rationalize the effect of network size on transcriptional output, we considered a simple combinatorial model in which all azide- and DBCO-modified monomers react with an equal probability to form covalent dimers. In this framework, a network composed of *m* DBCO monomers and *n* azide monomers can yield up to *m* × *n* distinct heterodimers, of which only one (D_act_) carries the correct pair of promoter halves to activate the mango template. The expected fraction of D_act_ within the total dimer pool is simply

$$f_{\mathrm{act}}=1/\left(m\times n\right)$$ 1

Thus, enlarging the network by adding new noncompetent monomer species is predicted to reduce the concentration of D_act_ ([D_act_]) inversely with the product *m* × *n*. Also, the fluorescence signal is proportional to the concentration of transcribed mango RNA, which in turn depends on the fraction of templates whose promoters are completed by D_act_. Assuming that the template activation follows a simple promoter-completion isotherm (as suggested by Figure 2f) characterized by a constant *K*_1/2_, the steady-state transcriptional output (i.e., fluorescence of the Mango aptamer) of the template is expected to scale as

$${\mathrm{fluorescence}}_{\mathrm{RNA}\text{\_}\mathrm{Mango}}\propto \left[D_{\mathrm{act}}\right]/\left(K_{1/2}+\left[D_{\mathrm{act}}\right]\right)$$ 2

When the experimentally measured end point fluorescence signals are plotted against the network size (for the specific symmetric case *m* = *n*), the data follow this predicted inverse trend and lie close to the theoretical curve obtained from the simple combinatorial model using a single effective *K*_1/2_ (3.5 ± 0.4 × 10^–8^ M, calculated from Figure 2f) (Figure 2i, dotted line). Since our goal in eq 2 is not to extract mechanistic cooperativity parameters but to obtain a single effective *K*_1/2_ for use in the network-size model, we retain the simple hyperbolic form in the main text for analytical clearness. The agreement between experimental results and the model confirms that the observed decrease in transcription upon network expansion primarily reflects the statistical dilution of the unique activator dimer within the many-to-many dimerization network, providing a direct, theory-guided design rule for programming transcriptional output by editing network size.

The many-to-many competitive DNA-based dimerization networks enable the programmable control of transcriptional output with different molecular inputs. To demonstrate this, we designed as inputs different DNA-based oligonucleotides that can bind to specific monomers and, by creating competitive duplexes, exclude them from the dimerization network (Figure 3a). More specifically, we used an initial network size of 22 (11 azide- and 11 DBCO-modified monomers) and then designed different inputs, each of which can specifically bind and exclude 2 azide- and 2 DBCO-modified monomers. This design represents a good compromise between achieving a large and quantifiable reduction in effective network size upon input addition and keeping the number of distinct input strands low to keep sequence orthogonality. This leads to a reduction in the size of the initial dimerization network (see Figure 3b) that follows the equation below:

reducednetworksize=initialnetworksize−4×ninputs

**Figure 3:**
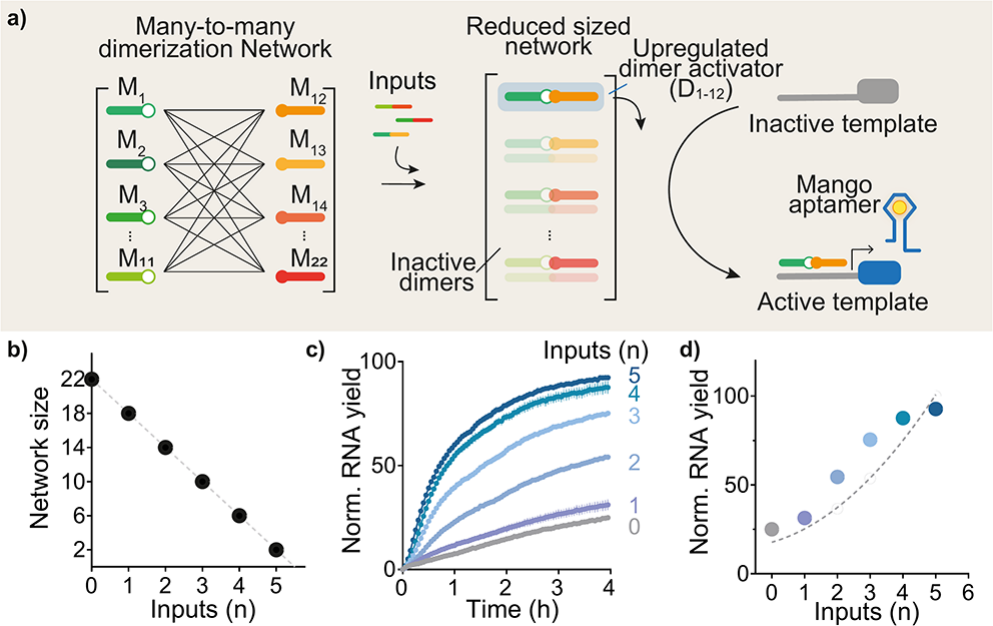
Design and validation of input-responsive many-to-many DNA dimerization transcription network. (a) Scheme of the original many-to-many dimerization network (network size = 22). Specific input strands populate a library of preformed, but inactive, heterodimers leading to an upregulation of the dimer activator that converts an inactive transcriptional template (gray) into its active form (blue). (b) Plot showing how the network size scales with the number of inputs added (each input sequesters 4 different monomers). (c) Time course experiments showing progressive increase in transcription efficiency with increasing number of input strands and (d) corresponding end point RNA yield versus number of inputs. The continuous line is not a fit, but the theoretical curve obtained by evaluating eq 1 and eq 2 using the experimentally determined *K*_1/2_ from Figure 2f. The experiments shown in this figure were performed as described in the caption of Figure 2. If present, inputs were used at a concentration of 1.0 μM and were introduced to the DBCO–DNA monomers immediately before adding the azide-DNA monomers. Error bars represent the standard deviation based on triplicate measurements.

As a result, the reduced network size leads to a more favorable reaction between the remaining monomers that ultimately leads to an upregulation of the yield of specific dimer outputs (Figure 3a). Quantitative fluorescence data of the mango aptamer reveal a strong correlation between the number of inputs and the magnitude of transcriptional signal, with increasing input numbers progressively upregulating the cell-free transcription over time (Figure 3c,d). These results confirm the ability of competitive DNA dimerization networks to integrate multiple molecular signals and convert them into tunable transcriptional outputs *in vitro*. Also in this case, modulation of cell-free transcription at different numbers of inputs agrees well with the theoretical yield (Figure 3d, dotted line).

The control of cell-free transcription through competitive dimerization networks is a versatile and highly orthogonal approach that enables the multiplexed operation of the same network for selective activation of distinct cell-free transcription templates. We demonstrated this by first combining the above-described many-to-many dimerization network with a new template activated by the same dimer output but encoding for a different light-up RNA aptamer (i.e., malachite green) and demonstrating a similar level of control and input–output computation (Figure S3). Similarly, we have adapted the dimerization network to upregulate a new dimer that could activate a template displaying the promoter domain of a different polymerase enzyme (i.e., SP6 polymerase) (Figure S4). We then proceeded to design a many-to-many dimerization network (network size = 24) that can produce, among others, two different dimer output activators, each of which activates only its matching transcription template, either for the malachite green aptamer^34^ (driven by T7 polymerase) or for the mango aptamer (driven by SP6 polymerase) (Figure 4a). By performing experiments in the absence and presence of two specific input sets, we demonstrate that template activation is directly coupled to input selection, as indicated by robust fluorescence outputs only when the corresponding input set is present (Figure 4b). This result highlights the capacity of competitive DNA dimerization networks to translate molecular input combinations into orthogonal, enzyme-driven gene expression events *in vitro*.

**Figure 4:**
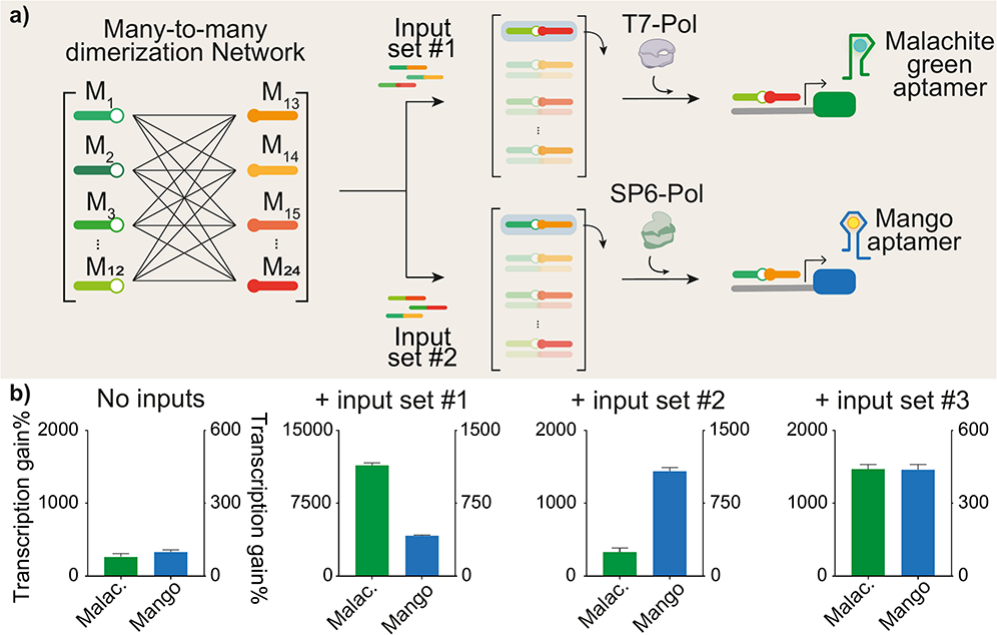
Competitive dimerization network for orthogonal control of multiple templates. (a) Scheme of the many-to-many dimerization network (network size = 24). Two input set configurations (input set #1 and input set #2) used to upregulate two different dimer activators each activating a different template-reporter pair in the same solution. (b) Transcription gain (%) obtained with the addition of different combinations of the two input sets. Last experiment shows the use of an input set (#3) that allows the upregulation of both dimer activators. The experiments shown in this figure were performed as described in the caption of Figure 2. In this case, the transcription solution contains both templates (each at 100 nM), T7 and SP6 polymerase and the mango aptamer dye (TO-1, 200 nM) and the malachite green dye (25 μM). Error bars represent the standard deviation based on triplicate measurements.

In a final set of experiments, we coupled the input-programmable dimerization network to a downstream CRISPR–Cas output module. To do so, we designed a many-to-many dimerization network (size = 22) so that it produces, among the many other dimers, an activator dimer that triggers a transcription template encoding for the specific guide RNA strand (crRNA) that activates Cas12a cleavage activity^35^ (Figure 5a). Downstream activation of Cas12a activity can be monitored in real time using a fluorophore-quencher labeled hairpin reporter.^36^ In the absence of any input, the many-to-many dimerization network distributes monomers over a large space of mostly inactive dimers, resulting in minimal formation of the specific dimer activator and thus minimal downstream activation of the Cas12a enzyme (Figure 5b, gray curve, 0 inputs). Upon addition of specific DNA inputs, the network is reduced to a smaller size, thus leading to an upregulated transcription of crRNA and thus to a higher downstream Cas12a nuclease activity (Figure 5b). More specifically, by adding inputs to gradually reduce the network size from 22 to 2, Cas collateral cleavage activity increases by an order of magnitude over the 8 h reaction window (Figure 5b-c).

**Figure 5:**
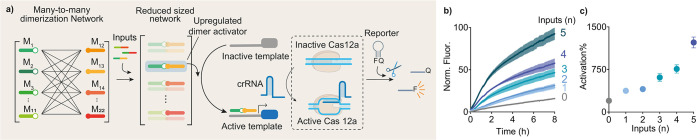
Cas12a collateral activity controlled by an input-programmed DNA dimerization network. (a) A many-to-many DNA dimerization network (network size = 22) that produces a pool of inactive dimers and a dimer activator. The dimer activator triggers the transcription of a Cas12a guide RNA (crRNA) switching Cas12a enzyme from an inactive to an active nuclease. (b) Time courses of reporter fluorescence and (c) end-point values for increasing numbers of inputs, illustrating graded Cas12a collateral activity as a function of network reduction and dimer activator upregulation. The experiments shown in this figure were performed in Tris-HCl 40 mM, MgCl_2_ 30 mM, DTT 10 mM, Spermidine 6 mM in DEPC water, pH 7. Each azide- and DBCO–DNA monomer was mixed at a concentration of 0.5 μM. If present, inputs were used at a concentration of 1.0 μM and were introduced to the DBCO–DNA monomers immediately before adding the azide-DNA monomers. At the end of each reaction, cell-free transcription was carried out as described in the caption of Figure 2 for 2 h. After cell-free transcription, the solution was diluted 1:10 in a buffer solution containing Cas12a enzyme and the reporter and fluorescence was measured continuously (see SI for details). Error bars represent the standard deviation based on triplicate measurements.

## Conclusions

Many natural gene regulatory systems make extensive use of promiscuous, many-to-many dimerization networks to implement complex input–output maps.^1–14^ Here, we demonstrated a minimal, DNA-encoded analogue of such architectures by rationally designing DNA-based dimerization networks that exploit the same type of competitive, many-to-many logic that underlies natural transcriptional decision-making. By using simple DNA monomers with orthogonal covalent handles (azide and DBCO) and wiring their dimerization products to incomplete promoter templates, we demonstrate that the size and composition of a dimerization network lead to predictable changes in cell-free transcriptional output. We also applied the same design principles with sequence-defined inputs to control the networks size and to selectively upregulate distinct transcription templates with different input combinations. By doing so, we demonstrated multiplexed, orthogonal activation of T7- and SP6-driven templates providing a route to realize competitive dimerization transcription logic. Coupling the dimerization layer to a CRISPR–Cas12a collateral cleavage module provides a single, concrete example of interfacing these networks with an RNA-controlled enzyme, converting input sets into amplified Cas-induced cleavage activity in a programmable fashion. These results define a core physical module whose behavior can be predicted from simple mass-action considerations of network size and input-controlled sequestration.

Together, these results show that synthetic DNA competitive dimerization networks can bridge concepts from systems chemistry, dynamic combinatorial libraries, and nucleic acid computation to implement addressable, input-responsive transcriptional regulation *in vitro*. Because our networks rely exclusively on sequence-encoded hybridization and robust SPAAC chemistry, they are inherently modular: new monomers, templates, and input strands can be added or exchanged without redesigning the core mechanism, while simple mass-action models capture the dependence of dimer yields and transcriptional outputs on network size and input number. For isolated regulation problems with fixed inputs and outputs, simpler solutions such as full-length activator oligonucleotides, toehold switches, or CRISPR-based regulators may be more direct.^39–41^ The distinctive advantage of the present strategy arises instead in settings where several outputs share a constrained monomer pool and where competition or mutual exclusion between outputs is a desired feature. Compared to other strand-displacement “computational” approaches, our competition-based strategy allows multiple potential activator dimers to share the same monomer pool, so that different input patterns of sequesters can redirect the dimer population and corresponding transcriptional outputs, in close analogy to competitive protein dimerization in transcription factor networks.

We emphasize that, in this work, we focus on single-output readouts of a shared dimerization network and we quantify how the transcriptional output of a selected template depends on network size and on input-mediated reduction of that size. Because all potential activator dimers come from a common monomer pool, our combinatorial model predicts that favoring one dimer necessarily reduces the availability of monomers for alternative dimers. However, a fully quantitative, multioutput demonstration of simultaneous upregulation of one template and down-regulation of others from the same pool is left as future work building on the present minimal platform.

Looking ahead, the ability to rationally program information via covalent dimerization networks suggests several routes for future development. Incorporating feedback motifs or cascades of dimer-controlled templates could yield higher-order behaviors such as adaptation, pulse generation, or winner-take-all selection. More broadly, the strategies introduced here provide the basis for building DNA-encoded dimerization networks that control not only reporter transcription but also enzyme activation, chemical synthesis, or materials assembly, moving from the foundational minimal subnetworks presented here toward more complex, application-oriented competitive circuits that more closely emulate the combinatorial control and decision-making capabilities of natural protein dimerization circuits.^37,38^

## Experimental Section

### Chemicals and Enzymes

Reagent-grade chemicals, DEPC-treated water, MgCl_2_, Tris-HCl, EDTA, NaCl, 1,4-dithiothreitol (DTT), and spermidine, were purchased from Sigma-Aldrich (Italy) and used without further purification. Nucleotide triphosphates (NTPs) were purchased from Jena Bioscience (Germany) and stored at −20 °C. T7 polymerase and RiboLock RNase inhibitors were purchased from Thermo Fisher Scientific (Waltham, MA, USA). Bacteriophage SP6 RNA Polymerase was purchased from New Engaland Biolabs (Ipswich, MA, USA). LbaCas12a from the *Lachnospiraceae bacterium* ND2006 is expressed as an N-terminal 6XHis-tagged fusion in *E. coli*. It was purchased from New England Biolabs (USA).

### Oligonucleotides

Oligonucleotides used in this work were synthesized, labeled, and HPLC-purified by Metabion International AG (Planegg, Germany) or Biomers (Germany) and used without further purification. All of the sequences are reported in the Supporting Information.

### Cell-free Transcription Experiments Using a Many-to-Many Dimerization Network

All the cell-free transcription experiments were carried out through fluorescence detection. First, the azide- and DBCO-modified monomers were mixed at an equimolar concentration of 1.0 mM in 50 μL of 1× IVT buffer (40 mM Tris-HCl, 30 mM MgCl_2_, 10 mM DTT, 6 mM spermidine, pH 7.0), at 25 °C for 24 h. At the end of the reaction, an aliquot (3 μL) was taken and added to a 27 μL solution of the transcription buffer (40 mM Tris-HCl, 30 mM MgCl_2_, 10 mM DTT, 6 mM spermidine, 3 mM nucleoside triphosphate (NTPs) mix, 3.0 U/μL T7 RNA polymerase (or 1.0 U/μL SP6 polymerase), 1 U/μL RiboLock, pH 7.0) containing the three strands forming the incomplete template (each at 100 nM) and the relevant dye (TO-1, 200 nM for mango aptamer or Malachite green dye, 25 μM for malachite green aptamer). The transcription reaction was allowed to proceed at 37 °C, and fluorescence signals were either measured in real time or at end point. Fluorescence signals were background-corrected using the corresponding no-dimer (or no-input) control. Normalized RNA yield in Figure 2h,i and Figure 3c,d was obtained by dividing the fluorescence values by the end point fluorescence value of the reference condition (network size = 2) set to 100%.

### Fluorescence Experiments

Fluorescence kinetic measurements were carried out on a Tecan F200pro plate reader using the top reading mode with black, flat bottom nonbinding 384-well plates and a 30 μL final volume. Detailed procedures employed are reported in the Supporting Information.

## Supplementary Material



## Notes

Oligonucleotide sequences used, additional experimental details and supplementary figures with additional networks (malachite green aptamer and SP6 polymerase), and control experiments (gel electrophoresis) (PDF)
