# Supplementary material for: Programmable DNA-Based Dimerization Networks for Dynamic and Competitive Control of Transcription
Source: J Am Chem Soc. 2026 Jul 20;148(30):31751–8. doi: 10.1021/jacs.6c02173 (PMC13450382; doi:10.1021/jacs.6c02173)
Supplement: Supplementary file 1 [file ja6c02173_si_001.pdf]

Supporting Information:

Programmable DNA-Based Dimerization Networks for Dynamic and Competitive Control of Transcription

Miriam Quattrociocchi, Simone Brannetti, Erica Del Grosso, Francesco Ricci *

1. EXPERIMENTAL PROCEDURES

1.1 DNA sequences

HPLC-purified oligonucleotides were purchased from Biomers (Germany), Biosearch Technologies (Risskov, Denmark) or Metabion International AG (Planegg, Germany) as lyophilized powder after HPLC purification. All oligonucleotides were dissolved in DEPC water at a concentration of 100 μM and stored at -20°C.

The sequences of the DBCO- and azide-modified monomers employed in this work are reported below:

Azide-modified and DBCO-modified DNA monomers (network size = 22) used in Figure 2, 3 and 5.

Name	Sequence 5' → 3'
**M_1_DBCO**	DBCO-ATG TAT TCC TCA
**M_2_DBCO**	DBCO-TGT TTG TTG AGT A
**M_3_DBCO**	DBCO-GTA TTG TCT GGT
**M_4_DBCO**	DBCO-GAG ATT GAT C
**M_5_DBCO**	DBCO-GGT AGT GGA ATG C
**M_6_DBCO**	ATA GAT CCT GAT AGC-DBCO
**M_7_DBCO**	GCC AAG TGG ATT GCG TC-DBCO
**M_8_DBCO**	ACA TGC GGA TTG-DBCO
**M_9_DBCO**	AGA GGA GGC T-DBCO
**M_{10}_DBCO**	ACC AGG AAG ATG T-DBCO
**M_{11}_DBCO**	DBCO-GAG ACC TAA C
**M_{12}_N_3**	TAT TAG TAT-C3-Azide
**M_{13}_N_3**	C3-Azide-TGT TTG TTG AGT A
**M_{14}_N_3**	C3-Azide- GTA TTG TCT GGT
**M_{15}_N_3**	C3-Azide- GAG ATT GAT C
**M_{16}_N_3**	C3-Azide- ACC AGG AAG ATG T
**M_{17}_N_3**	ATA GAT CCT GAT AGC-C3-Azide
**M_{18}_N_3**	GCC AAG TGG ATT GCG TC-C3-Azide
**M_{19}_N_3**	ACA TGC GGA TTG-C3-Azide
**M_{20}_N_3**	AGA GGA GGC T-C3-Azide
**M_{21}_N_3**	GGT AGT GGA ATG C-C3-Azide
**M_{22}_N_3**	C3-Azide-GAG ACC TAA C

The monomers denoted as M_1_DBCO and M_{12}_N3 are those forming the activator dimer that will activate the incomplete template for mango aptamer. Input strands are 14–18 nt

single-stranded DNAs complementary to unique regions of their target monomers, with GC content between 40–60% to ensure strong binding at 25 °C in the SPAAC buffer while minimizing secondary structure and cross-hybridization. Orthogonality and absence of significant secondary structure were verified using NUPACK.

The sequences of the input strands are reported below:

Input DNA strands (Network size =22)

**Name**	**Sequence 5' → 3'**
**Input$_1$**	GTT AGG TCT CGC TAT CAG GAT CTA T
**Input$_2$**	TAC TCA ACA AAC AGA CGC AAT CCA CTT GGC
**Input$_3$**	ACC AGA CAA TAC CAA TCC GCA TGT
**Input$_4$**	ACA TCT TCC TGG TGC ATT CCA CTA CC
**Input$_5$**	GAT CAA TCT CAG CCT CCT CT

Azide-modified and DBCO-modified DNA monomers (network size = 24) used in Figure 4.

**Name**	**Sequence 5' → 3'**
**M$_1$_DBCO**	DBCO-ATG TAT TCC TCA
**M$_2$_DBCO**	DBCO-ACT TTC ATT TGC
**M$_3$_DBCO**	DBCO-GTA TTG TCT GGT
**M$_4$_DBCO**	DBCO-GAG ATT GAT C
**M$_5$_DBCO**	DBCO-GGT AGT GGA ATG C
**M$_6$_DBCO**	ATA GAT CCT GAT AGC-DBCO
**M$_7$_DBCO**	GCC AAG TGG ATT GCG TC-DBCO
**M$_8$_DBCO**	ACA TGC GGA TTG-DBCO
**M$_9$_DBCO**	AGA GGA GGC T-DBCO
**M$_{10}$_DBCO**	ACC AGG AAG ATG T-DBCO
**M$_{11}$_DBCO**	DBCO-GAG ACC TAA C
**M$_{12}$_DBCO**	DBCO-TGT TTG TTG AGT A
**M$_{13}$_N$_3$**	TAA ATG TAT-C3-Azide
**M$_{14}$_N$_3$**	TAT TAC TTT-C3-Azide
**M$_{15}$_N$_3$**	C3-Azide-TGT TTG TTG AGT A
**M$_{16}$_N$_3$**	C3-Azide- GTA TTG TCT GGT
**M$_{17}$_N$_3$**	C3-Azide- GAG ATT GAT C
**M$_{18}$_N$_3$**	C3-Azide- ACC AGG AAG ATG T
**M$_{19}$_N$_3$**	ATA GAT CCT GAT AGC-C3-Azide
**M$_{20}$_N$_3$**	GCC AAG TGG ATT GCG TC-C3-Azide
**M$_{21}$_N$_3$**	ACA TGC GGA TTG-C3-Azide
**M$_{22}$_N$_3$**	AGA GGA GGC T-C3-Azide
**M$_{23}$_N$_3$**	GGT AGT GGA ATG C-C3-Azide
**M$_{24}$_N$_3$**	C3-Azide-GAG ACC TAA C

The monomers denoted as M_1_DBCO and M_{13}_N_3 are those forming the activator dimer that will activate the incomplete template for mango aptamer driven by the SP6 polymerase. The monomers denoted as M_2_DBCO and M_{14}_N_3 are those forming the activator dimer that will activate the incomplete template for malachite green aptamer driven by the T7 polymerase.

The sequences of the input strands are reported below:

Input set #1 (Network size = 24)

This input set was used to upregulate the formation of the activator dimer that will activate the incomplete template for malachite green aptamer driven by the T7 polymerase.

**Name**	**Sequence 5' → 3'**
**$Input_1$**	GTT AGG TCT CGC TAT CAG GAT CTA T
**$Input_2$**	TAC TCA ACA AAC AGA CGC AAT CCA CTT GGC
**$Input_3$**	ACC AGA CAA TAC CAA TCC GCA TGT
**$Input_4$**	ACA TCT TCC TGG TGC ATT CCA CTA CC
**$Input_5$**	GAT CAA TCT CAG CCT CCT CT
**$Input_6$**	TGA GGA ATA CAT ATA CAT TTA

Inputs from 1 to 5 are able to sequester 2 azide- and 2 DBCO-monomers while $input_6$ is only able to sequester 1 azide- and 1 DBCO-monomer.

Input set #2 (Network size = 24)

This input set was used to upregulate the formation of the activator dimer that will activate the incomplete template for mango aptamer driven by SP6 polymerase.

**Name**	**Sequence 5' → 3'**
**$Input_1$**	GTT AGG TCT CGC TAT CAG GAT CTA T
**$Input_2$**	TAC TCA ACA AAC AGA CGC AAT CCA CTT GGC
**$Input_3$**	ACC AGA CAA TAC CAA TCC GCA TGT
**$Input_4$**	ACA TCT TCC TGG TGC ATT CCA CTA CC
**$Input_5$**	GAT CAA TCT CAG CCT CCT CT
**$Input_7$**	ATA ATG AAA TGA AAG TAA ACG

Inputs from 1 to 5 are able to sequester 2 azide- and 2 DBCO-monomers while $input_7$ is only able to sequester 1 azide- and 1 DBCO-monomer.

Input set #3 (Network size = 24)

This input set was used to upregulate the formation of both activator dimers that will activate the incomplete template for mango and malachite green aptamers.

**Name**	**Sequence 5' → 3'**
**Input$_1$**	GTT AGG TCT CGC TAT CAG GAT CTA T
**Input$_2$**	TAC TCA ACA AAC AGA CGC AAT CCA CTT GGC
**Input$_3$**	ACC AGA CAA TAC CAA TCC GCA TGT
**Input$_4$**	ACA TCT TCC TGG TGC ATT CCA CTA CC
**Input$_5$**	GAT CAA TCT CAG CCT CCT CT

Inputs from 1 to 5 are able to sequester 2 azide- and 2 DBCO-monomers.

The sequences of the transcription template encoding for the mango aptamer and driven by T7 polymerase (Figure 2 and 3) are reported below:

**Name**	**Sequence 5' → 3'**
Non-Template strand	**TGA GGA ATA CAT ATA** CTA ATA CGA CTC ACT ATA *GGG CAC GTA CGA AGG AAG GTT TGG TAT GTG GTA TAT TCG TAC GTG C*
Incomplete Template strand	*GCA CGT ACG AAT ATA CCA CAT ACC AAA CCT TCC TTC GTA CGT GCC C*TA TAG TGA GTC G
Blocker strand	**TAT ATG TAT TCC TCA**

In the sequences above the underlined nucleotides represent the 17-nt promoter portion of the template recognized by T7 polymerase. The incomplete template strand has 5-nt portion missing in the reporter domain that prevents efficient transcription. The nucleotides in italics represent the portion that encodes for the 46-nt mango aptamer.

The 15-nt blocker strand is designed to bind to the non-template strand 6-nt upstream of the promoter domain to avoid any undesired reaction between unreacted monomers and the non-template strand.

The sequences of the transcription template encoding for the malachite green aptamer and driven by T7 polymerase (Figure 4) are reported below:

**Name**	**Sequence 5' → 3'**
Non-Template strand	**GCA AAT GAA AGT AAA** GTA ATA CGA CTC ACT ATA *GGG ATC CCG ACT GGC GAG AGC CAG GTA ACG AAT GGA TC*
Incomplete Template strand	*GAT CCA TTC GTT ACC TGG CTC TCG CCA GTC GGG ATC CC*T ATA GTG AGT CG
Blocker strand	**TTT ACT TTC ATT TGC**

In the sequences above the underlined nucleotides represent the 17-nt promoter portion of the template recognized by T7 polymerase. The incomplete template strand has 5-nt portion missing in the reporter domain that prevents efficient transcription. The nucleotides in italics represent the portion that encodes for the 38-nt malachite green aptamer.

The 15-nt blocker strand is designed to bind to the non-template strand 6-nt upstream of the promoter domain to avoid any undesired reaction between unreacted monomers and the non-template strand.

The sequences of the Transcription template encoding for the mango aptamer and driven by SP6 polymerase (Figure 4) are reported below:

**Name**	**Sequence 5' → 3'**
Non-Template strand	**TGA GGA ATA CAT ATA** CAT TTA GGT GAC ACT ATA *GGG GCA CGT ACG AAG GAA GGT TTG GTA TGT GGT ATA TTC GTA CGT GC*
Incomplete Template strand	*GCA CGT ACG AAT ATA CCA CAT ACC AAA CCT TCC TTC GTA CGT GCC* CCT ATA GTG TCA CC
Blocker strand	**TAT ATG TAT TCC TCA**

In the sequences above the underlines nucleotides represent the 17-nt promoter portion of the template recognized by SP6 polymerase. The incomplete template strand has 5-nt portion missing in the reporter domain that prevents efficient transcription. The nucleotides in italics represent the portion that encodes for the 46-nt mango aptamer.

The 15-nt blocker strand is designed to bind to the non-template strand 6-nt upstream of the promoter domain to avoid any undesired reaction between unreacted monomers and the non-template strand.

The sequences of the transcription template encoding for the guide RNA of the Cas12a enzyme and driven by T7 polymerase (Figure 5) are reported below:

**Name**	**Sequence 5' → 3'**
Non-Template strand	**TGA GGA ATA CAT ATA** CTA ATA CGA CTC ACT ATA *GGG TAA TTT CTA CTA AGT GTA GAT GTC TAC ACA TGG CTA AAT CT*

Incomplete Template strand	AGA TTT AGC CAT GTG TAG ACA TCT ACA CTT AGT AGA AAT TA CCC <u>TAT AGT GAG TCG</u>
Blocker strand	**TAT ATG TAT TCC TCA**

In the sequences above the underlined nucleotides represent the 17-nt promoter portion of the template recognized by T7 polymerase. The incomplete template strand has 5-nt portion missing in the reporter domain that prevents efficient transcription. The nucleotides in italics represent the portion that encodes for the guide RNA aptamer.

The 15-nt blocker strand is designed to bind to the non-template strand 6-nt upstream of the promoter domain to avoid any undesired reaction between unreacted monomers and the non-template strand.

For Cas12a cleavage experiments we also used the following reporter:

**Name**	**Sequence 5' → 3'**
Cas12a Reporter	(6-Fam) - CTC TCA TTT TTA GAG AG - (BHQ1)

1.2 Control of Cas12a collateral cleavage activity using a many-to-many dimerization network.

Time-course experiments in Figure 5, were performed as reported above using the incomplete template encoding for the guide RNA. After transcription reaction (2 hours) an aliquot (3 μL) of the reaction was taken and added to a 27 μL buffer solution (10 nM Tris-HCl, 50 mM NaCl, 10 mM $MgCl_2$, pH 7.8) containing 2 nM LbaCas12a and 100 nM of the DNA reporter. The cleavage reaction was incubated at 37° C and the fluorescence intensity was recorded over time.

1.3 Fluorescence measurements

Time-course fluorescence measurements of cell-free transcription reactions and Cas12a reactions were carried out on a Tecan F200pro plate reader using the top reading mode with black, flat bottom non-binding 384-well plates and a 30 µL final volume. The working wavelengths were set to λ_{ex} = 485 (± 9) nm and λ_{em} = 535 (± 20) nm for the templates transcribing the mango aptamer (Figure 2 and 3). For the templates transcribing the malachite Green aptamer (Figure 4 and S2) the wavelengths were λ_{ex} = 625 (±9) nm and λ_{em} = 660 (± 20) nm. For Cas12a cleavage experiments (Figure 5) that used the fluorescently labelled DNA reporter with 6-FAM the working wavelengths were set to λ_{ex} = 490 (±9) nm and λ_{em} = 525 (±20) nm.

The emission spectrum (Figure 2d) of the mango dye in the presence and absence of the aptamer was measured using a Cary Eclipse Fluorescence Spectrophotometer (Agilent Technologies). The excitation wavelength was 485 nm, and the emission was recorded between 520 and 600 nm.

1.4 Gel Electrophoresis

Polyacrylamide gels (18%) were run at room temperature in running 1x TAE buffer (Tris base 40 mM, 1 mM EDTA, 20 mM acetic acid, pH 8.0) at 80 V. Sample volumes of 10 µl were combined with 1 µl of 6× DNA Loading Dye followed by direct loading into the gel. After the run, the gels were stained with SYBR Gold (invitrogen, Thermo Fisher Scientific) for 20 minutes. Gels were scanned by a ChemiDoc MP imaging system (Bio-Rad) and imaged.

1.5 Ion exchange chromathography

Ion exchange chromathography showing the formation of the covalent DNA dimer product from the two DNA monomer precursors was performed using the ProFire instrument with UV-detector set at a wavelength of 260 nm (Dynamic Biosensors, Germany). The samples of the azide-modified and DBCO-modified precursors were diluted at 1.0 µM in a 150 µL 1x IVT buffer (40 mM Tris-HCl, 30 mM $MgCl_2$, 10 mM DTT, 6 mM Spermidine, pH 7.0). For the covalent DNA dimer product characterization, the azide- and DBCO-modified monomers were mixed at an equimolar concentration of 1.0 µM in a 150 mL 1x IVT buffer (40 mM Tris-HCl, 30 mM $MgCl_2$, 10 mM DTT, 6 mM Spermidine, pH 7.0), at 25°C for 24 hours. After preparation, all samples were injected into the instrument separately. The runs were recorded for approximately 15 minutes.

2. SUPPORTING FIGURES

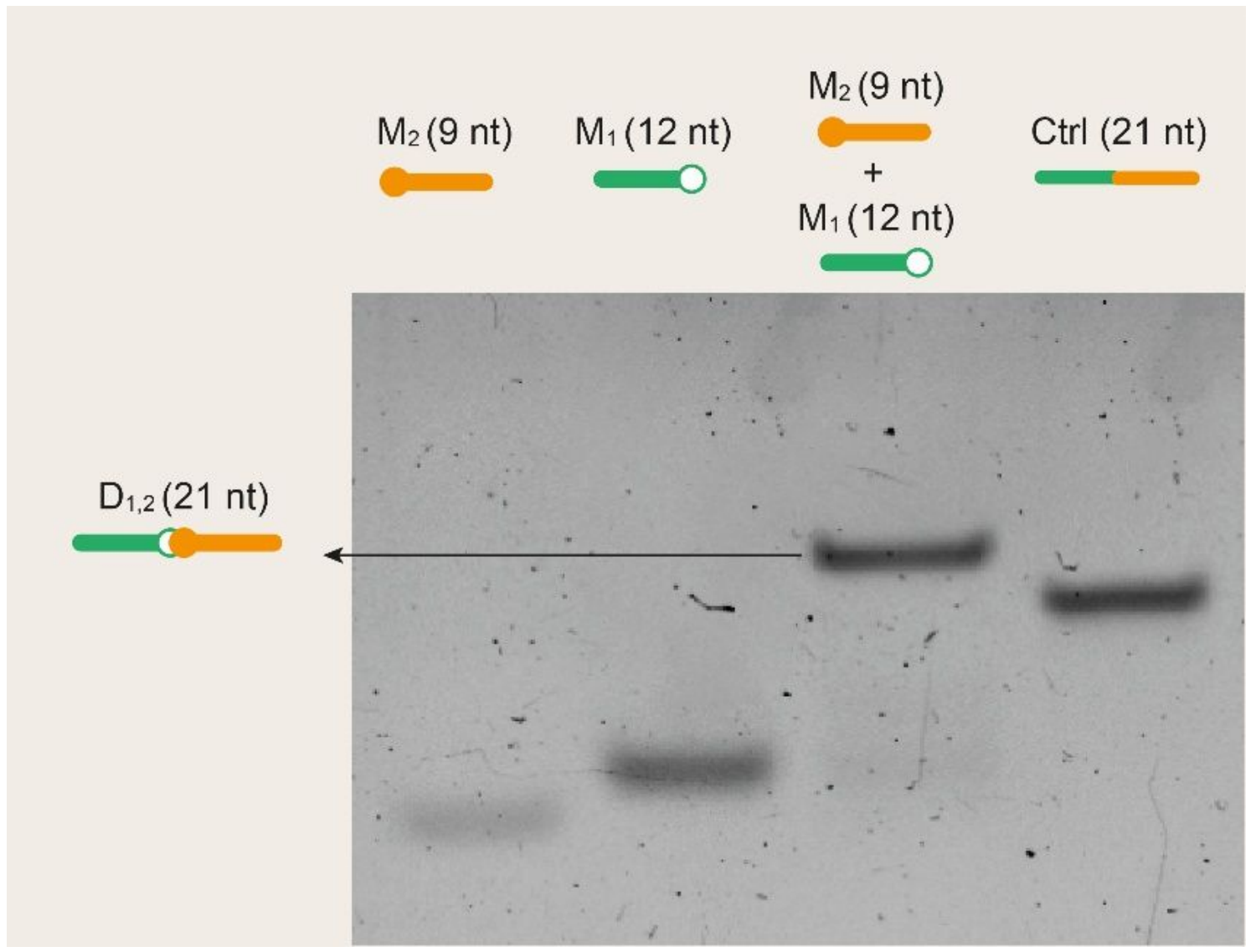

Figure S1. Polyacrylamide gel (18%) electrophoresis experiment to confirm successful dimer formation. From left to right the following reagents were added to the gel: M2 monomer, M1 monomer, M2 + M1 let to react for 24 hours, control strand with the same length as the dimer. Gel was run at room temperature in running 1x TAE buffer (Tris base 40 mM, 1 mM EDTA, 20 mM acetic acid, pH 8.0) at 80 V. Sample volumes of 10 µl were combined with 1 µl of 6× DNA Loading Dye followed by direct loading into the gel.

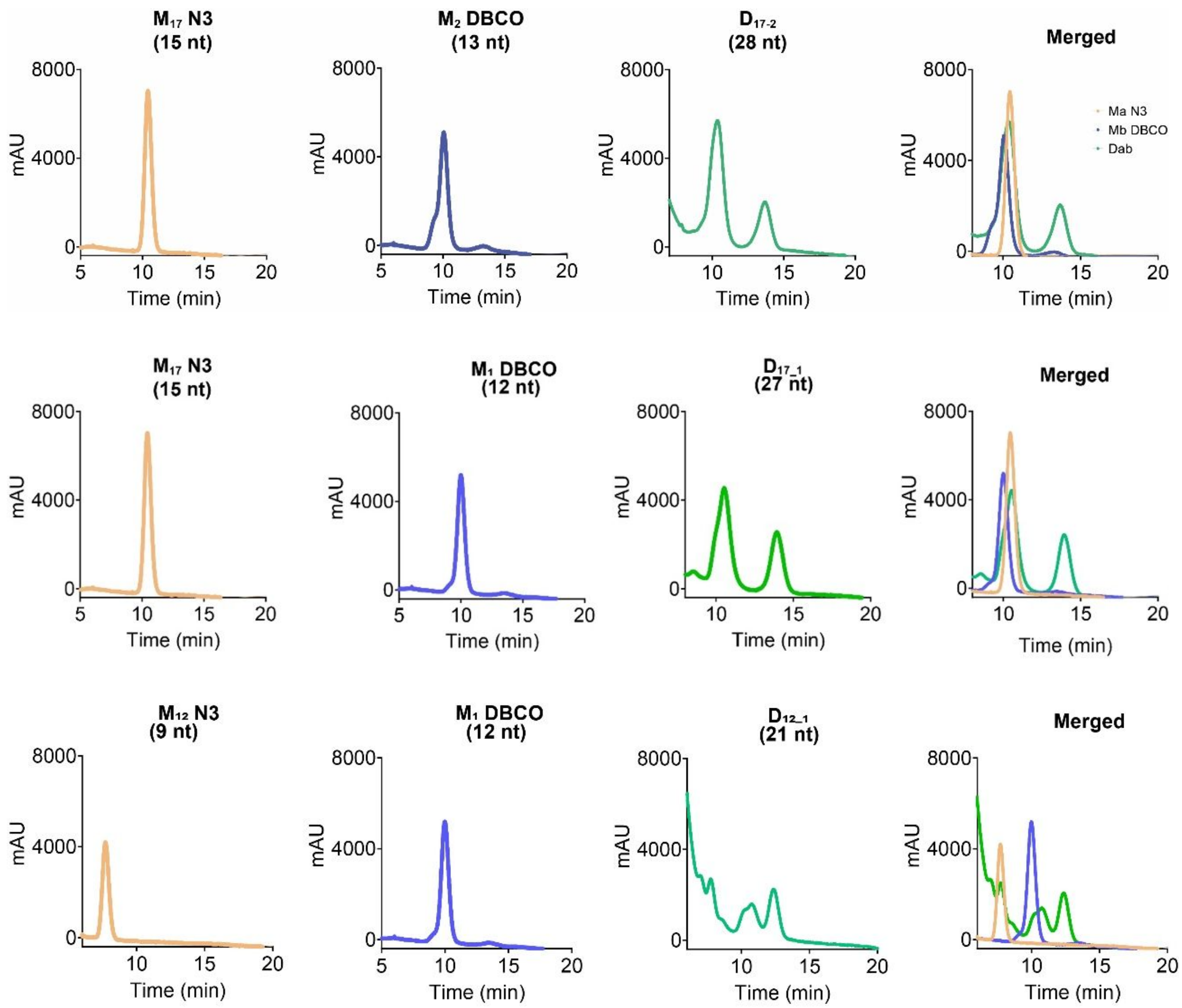

M_N$_3$	M_DBCO	Yield %
M17 (15 nt)	M2 (13 nt)	24.07%
M17 (15 nt)	M1 (12 nt)	30.04%
M12 (9 nt)	M1 (12 nt)	27.87%

Figure S2: Ion-exchange chromatography for three different pairs of monomers and three different dimers before (orange and blue traces) and after (green trace) SPAAC reaction. Reactions were performed as reported in Figure 2. Briefly, each azide- and DBCO-DNA monomer was mixed at a concentration of 0.5 μM in a transcription buffer solution (Tris-HCl 40 mM, $MgCl_2$ 30 mM, DTT 10 mM, Spermidine 6 mM, pH 7.0) and let to react for 24 hours. The yield of each dimer was calculated by dividing the area of the peak corresponding to the product by the sum of the areas corresponding to the monomers. In the green traces (after the reaction) the peak of the product corresponds to the one at t_{ret} of ca. 15 minutes. The following monomers were used in these experiments: Top: M17_N3 (15 nt) + M2_DBCO (13 nt); Center: M17_N3 (15 nt) + M1_DBCO (12 nt); Bottom: M12_N3 (9 nt) + M1_DBCO (12 nt). The calculated yield for each experiment is reported in the table.

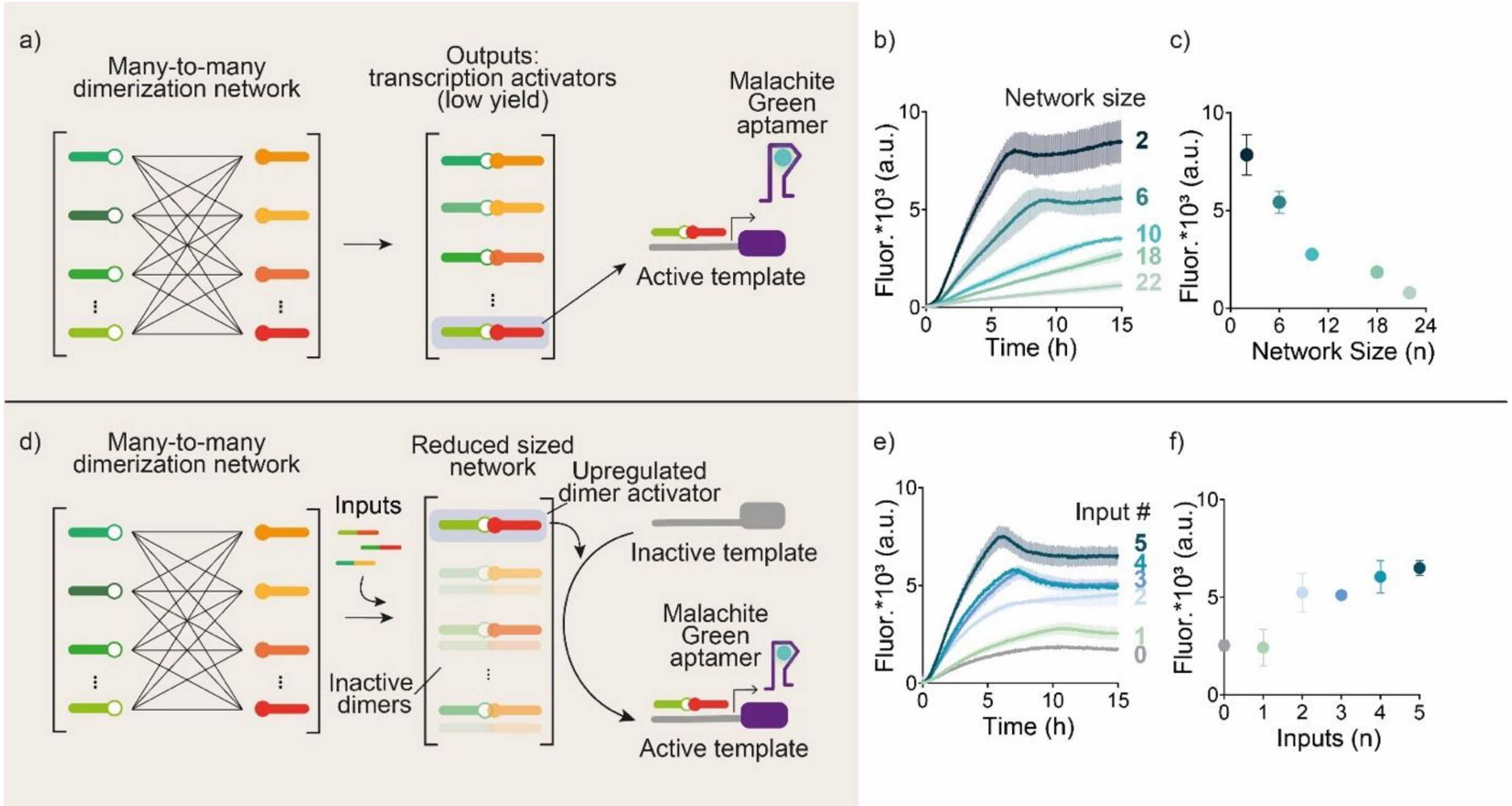

Figure S3. Many-to-many dimerization network for transcription control of malachite green aptamer. (a) Scheme of the many-to-many DNA dimerization network with a dimer activating the malachite green template. (b) Time course fluorescent experiments for networks of increasing size (from 2 to 22). (c) Endpoint fluorescent signals as a function of network size. (d) Schematic of the input-responsive many-to-many dimerization network. (e) Time course experiments showing progressive increase in transcription efficiency with increasing number of input strands and (f) corresponding endpoint signals versus number of inputs. The experiments shown in this figure were performed in IVT buffer (Tris-HCl 40 mM, $MgCl_2$ 30 mM, DTT 10 mM, Spermidine 6 mM in DEPC water, pH 7.0). Each azide- and DBCO-DNA monomer was mixed at a concentration of 0.5 μM. If present, inputs were used at a concentration of 1.0 μM and were introduced to the DBCO-DNA monomers immediately before adding the azide-DNA monomers. At the end of the reaction, the network solution was diluted 1:10 in a transcription buffer solution (Tris-HCl 40 mM, $MgCl_2$ 30 mM, DTT 10 mM, Spermidine 6 mM, nucleoside triphosphate (NTPs) mix 3 mM, T7 RNA polymerase 3.0 U/μL, RiboLock 1 U/μL, pH 7.0) containing the incomplete template (100 nM) and the relevant dye (25 mM for malachite green aptamer). The transcription reaction was allowed to proceed at 37 °C and fluorescent intensity was measured continuously. Error bars represent the standard deviation based on triplicate measurements.

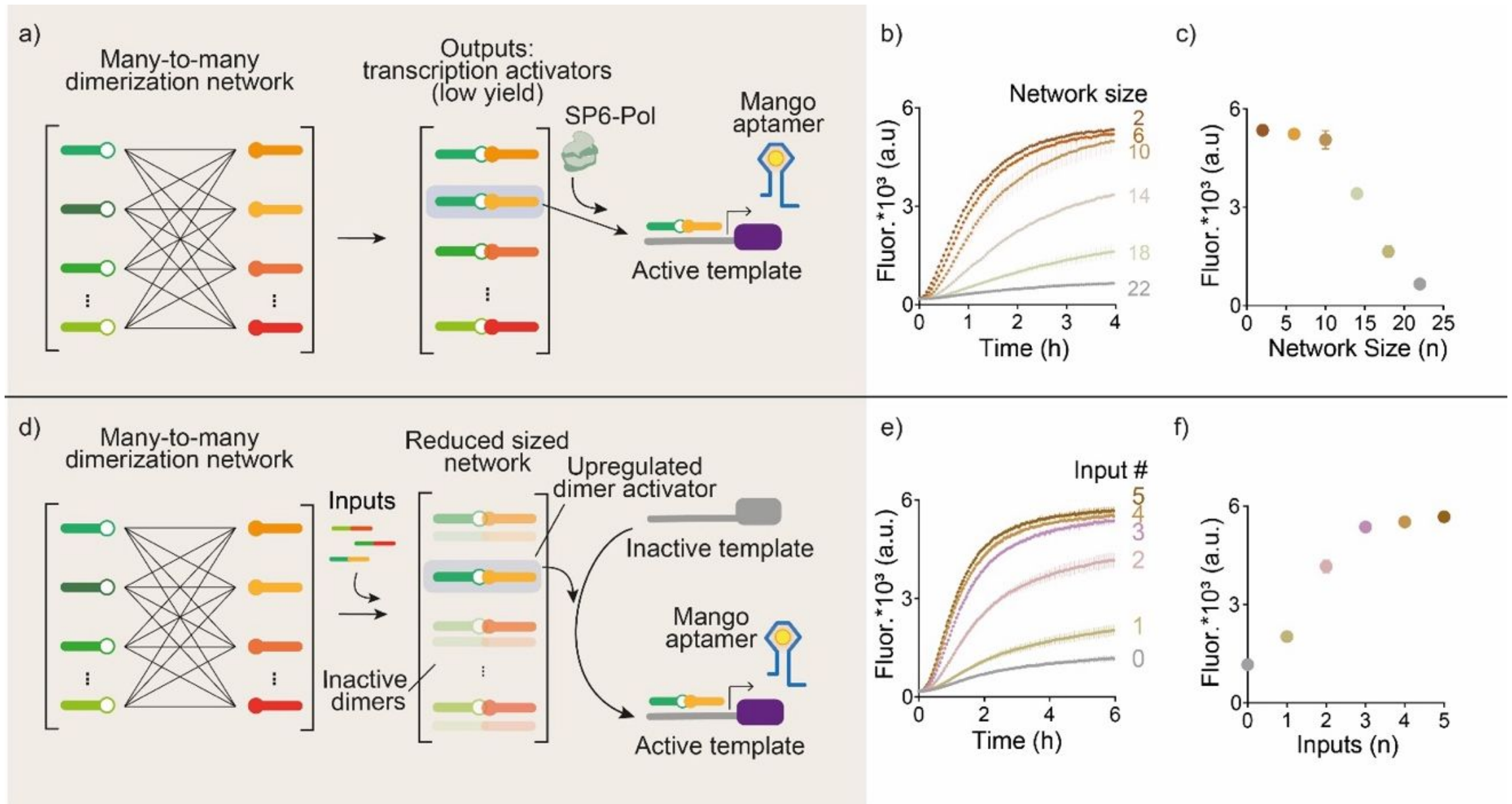

Figure S4. Many-to-many dimerization network for transcription control of mango aptamer using SP6 polymerase. (a) Scheme of the many-to-many DNA dimerization network with a dimer activating the mango aptamer template recognized by SP6 polymerase. (b) Time course fluorescent experiments for networks of increasing size (from 2 to 22). (c) Endpoint fluorescent signals as a function of network size. (d) Schematic of the input-responsive many-to-many dimerization network. (e) Time course experiments showing progressive increase in transcription efficiency with increasing number of input strands and (f) corresponding endpoint signals versus number of inputs. The experiments shown in this figure were performed in IVT buffer (Tris-HCl 40 mM, $MgCl_2$ 30 mM, DTT 10 mM, Spermidine 6 mM in DEPC water, pH 7.0). Each azide- and DBCO-DNA monomer was mixed at a concentration of 0.5 µM. If present, inputs were used at a concentration of 1.0 µM and were introduced to the DBCO-DNA monomers immediately before adding the azide-DNA monomers. At the end of the reaction, the network solution was diluted 1:10 in a transcription buffer solution (Tris-HCl 40 mM, $MgCl_2$ 30 mM, DTT 10 mM, Spermidine 6 mM, nucleoside triphosphate (NTPs) mix 3 mM, SP6 RNA polymerase 1 U/µL, RiboLock 1 U/µL, pH 7.0) containing the incomplete template (100 nM) and the mango aptamer dye (TO-1, 200 nM) The transcription reaction was allowed to proceed at 37 °C and fluorescent intensity was measured continuously. Error bars represent the standard deviation based on triplicate measurements.
